# Comparison of the Concordance of Allergic Diseases between Monozygotic and Dizygotic Twins: A Cross-Sectional Study Using KoGES HTS Data

**DOI:** 10.3390/jpm13050721

**Published:** 2023-04-25

**Authors:** Eun Jae Lee, Joo-Hee Kim, Hyo Geun Choi, Ho Suk Kang, Hyun Lim, Ji Hee Kim, Seong-Jin Cho, Eun Sook Nam, Ha Young Park, Nan Young Kim, Mi Jung Kwon

**Affiliations:** 1 College of Nursing, Ewha Womans University, Seoul 03760, Republic of Korea; soa5070@naver.com; 2 Department of Medicine, Division of Pulmonary, Allergy, and Critical Care Medicine, Hallym University Sacred Heart Hospital, Hallym University College of Medicine, Anyang 14068, Republic of Korea; luxjhee@gmail.com; 3 Suseo Seoul E.N.T. Clinic and MD Analytics, 10, Bamgogae-ro 1-gil, Gangnam-gu, Seoul 06349, Republic of Korea; mdanalytics@naver.com; 4 Division of Gastroenterology, Department of Internal Medicine, Hallym University Sacred Heart Hospital, Hallym University College of Medicine, Anyang 14068, Republic of Korea; hskang76@hallym.or.kr (H.S.K.); hlim77@hallym.or.kr (H.L.); 5 Department of Neurosurgery, Hallym University Sacred Heart Hospital, Hallym University College of Medicine, Anyang 14068, Republic of Korea; kimjihee.ns@gmail.com; 6 Department of Pathology, Kangdong Sacred Heart Hospital, Hallym University College of Medicine, Seoul 05355, Republic of Korea; apilas@hanmail.net (S.-J.C.); esnam@kdh.or.kr (E.S.N.); 7 Department of Pathology, Busan Paik Hospital, Inje University College of Medicine, Busan 47392, Republic of Korea; 8 Hallym Institute of Translational Genomics and Bioinformatics, Hallym University Medical Center, Anyang 14068, Republic of Korea; honeyny78@gmail.com; 9 Department of Pathology, Hallym University Sacred Heart Hospital, Hallym University College of Medicine, Anyang 14068, Republic of Korea

**Keywords:** dizygotic twins, monozygotic twins, allergic disease, asthma, allergic rhinitis, atopic dermatitis, allergic conjunctivitis, genetic factors, environmental factors

## Abstract

Several epidemiological studies have demonstrated that genetic and environmental factors contribute to the development of allergic diseases. However, there is limited information on these factors in the Korean population. This study investigated the importance of genetic and environmental factors in allergic diseases, such as allergic rhinitis, asthma, allergic conjunctivitis, or atopic dermatitis, by comparing the disease incidence in Korean adult monozygotic and dizygotic twins. This cross-sectional study utilized data from 1296 twin pairs, including 1052 monozygotic and 244 dizygotic twins, aged over 20 years, from the Korean Genome and Epidemiology Study (2005–2014). The study utilized binomial and multinomial logistic regression models to compute odds ratios of disease concordance. The concordance rate (92%) of the presence or absence of atopic dermatitis in monozygotic twins was slightly higher than that in dizygotic twins (90.2%), which only had a borderline significance (*p* = 0.090). The concordance rates of other allergic diseases within monozygotic twins were lower compared to dizygotic twins (asthma, 94.3% vs. 95.1%; allergic rhinitis, 77.5% vs. 78.7%; allergic conjunctivitis, 90.6% vs. 91.8%), of which the differences were not statistically significant. Monozygotic twins had a higher proportion of cases in which both siblings had allergic diseases than dizygotic twins (asthma, 1.1% vs. 0.0%; allergic rhinitis, 6.7% vs. 3.3%; atopic dermatitis, 2.9% vs. 0.0%; allergic conjunctivitis, 1.5% vs. 0.0%), of which the differences were also not statistically significant. In conclusion, our results appear to indicate the relative importance of environmental factors over genetic factor in the development of allergic diseases in Korean adult monozygotic twins.

## 1. Introduction

Allergic diseases, such as allergic rhinitis, asthma, allergic conjunctivitis, or atopic dermatitis, encompass a broad range of clinically observable phenotypes and distinct immunological and molecular mechanisms, forming the spectrum of the allergic march [1]. The allergic spectrum is a proposed sequence of atopic disease progression, wherein infants with atopic dermatitis may later develop asthma and subsequently experience allergic rhinitis or conjunctivitis [2]. These allergic disorders are prevalent globally, with 300 million individuals suffering from asthma [3], 400 million experiencing allergic rhinitis [3], and 6–30% of the general population being impacted by allergic conjunctivitis [4].

The development of allergic disorders is influenced by genetic, epigenetic, and environmental factors that result in inappropriate immune responses [5]. The prevalence rates of asthma and allergic rhinitis have leveled off in Western countries but continue to rise in many Asian countries [6,7]. Allergic diseases are generally less common in the Asia-Pacific region [8]; however, prevalence rates differ among Asian countries due to variations in development and environmental exposure [7]. In Korea, the prevalence of asthma and atopic dermatitis decreases with age, while the prevalence of allergic rhinitis and allergic conjunctivitis increases with age [9], which differs from patterns observed in other Western and Asian countries. Genetic variants identified via genome-wide association studies have a minor impact on the development of allergic diseases [8,10,11,12], although family history is deemed crucial, as having a sibling with asthma increases the risk of asthma (odds ratio [OR] 5.68) [13]. Environmental factors play a significant role in the development of allergic diseases [8]. However, there is a lack of information on the relative significance of genetic and environmental factors that contribute to allergic diseases in the Korean population, making it important to assess the current genetic and environmental influences on allergic diseases in Korea to identify risk factors and prevent these diseases, especially as Korea undergoes a dynamic transition from a developing to a developed country [14,15].

Twin studies are valuable tools for investigating the roles of genetic and environmental factors in the development of allergic diseases. These studies allow for the determination of the relative contributions of these factors in causing allergic diseases [16]. Monozygotic twins are often used in such studies because they possess a nearly identical genetic makeup, making it easier to investigate the impact of environmental factors on the development of complex diseases and physical traits [17]. The similarity between monozygotic twins is more influenced by genetic factors than that between dizygotic twins, whereas environmental factors may account for similar levels of dissimilarity between two individuals within a twin pair [16]. Despite the potential benefits of twin studies, only a few studies have comprehensively analyzed the genetic and environmental influences on allergy-related traits using validated twin cohort data. Some studies have compared concordance rates for asthma between monozygotic and dizygotic twins [16,18]; however, fewer studies have compared rates of other allergic diseases, such as allergic rhinitis, atopic dermatitis, or allergic conjunctivitis. Furthermore, clinical trials have been conducted to understand the differences in genetic and phenotypic characteristics between twin siblings with and without allergic diseases (ClinicalTrials.gov Identifier: NCT01613885) [19]. Conversely, due to potential shared risk factors and reciprocal relationships among various allergic diseases, it is essential to conduct further studies that control for potential mutual confounding factors.

Therefore, the objective of this study was to examine the impact of environmental factors on the genetic component of allergic diseases, such as asthma, allergic rhinitis, atopic dermatitis, and allergic conjunctivitis, among twins. We analyzed the occurrence and similarity of allergic diseases in monozygotic twins (indicative of genetic influence) and dizygotic twin pairs (reflecting the more significant contribution of environmental factors over genetic influence), while also accounting for lifestyle factors.

## 2. Materials and Methods

### 2.1. Study Population and Data Collection

The ethics committee of Hallym University approved the study (No. 2021-03-004). The institutional review board waived the requirement for obtaining written consent. The cohort study drew information collected between 2005 and 2014 from the Korean Genome and Epidemiology Study (KoGES, http://www.nih.go.kr/NIH/eng/main.jsp, accessed on 1 June 2022), which commenced in 2001 [20,21,22,23]. The data used was taken from the KoGES Healthy Twin Study (HTS), which was a part of the KoGES consortium (baseline and follow-up data from 2005 to 2013 and from 2008 to 2014, respectively) [23]. The KoGES HTS is an ongoing multicenter cohort study of city dwellers aged ≥20 years initiated in 2005 [24]. Zygosity was determined at the start using a questionnaire with >90% precision and a genetic examination, including 16 short tandem repeat markers (AmpFlSTR Identifier Kit; Perkin Elmer, Waltham, MA, USA) [25]. Of the participants who attended the baseline examination (2005–2013), two-thirds were monitored for follow-up (2008–2014) and their medical histories were kept up to date.

### 2.2. Participant Selection

**Figure 1:**
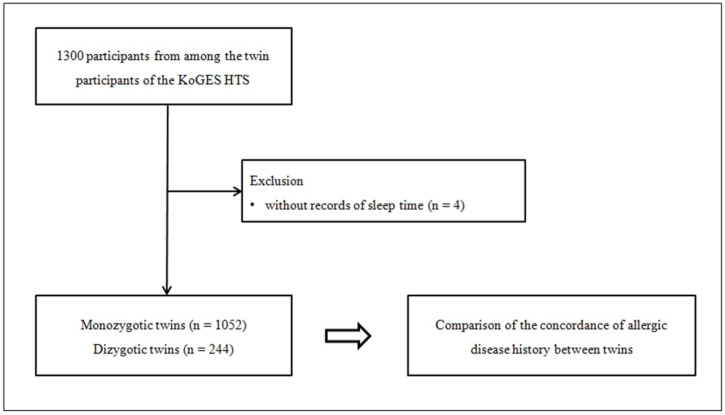
This study aimed to compare the concordance of allergic disease history between 1052 monozygotic and 244 dizygotic twins. The study design involved examining both types of twins to identify similarities and differences in the incidence and presentation of allergic diseases.

For this cross-sectional study, we chose 1300 twins from the KoGES HTS database. We eliminated those who did not have records of sleep time, leaving 1052 monozygotic (526 pairs) and 244 dizygotic twins (122 pairs) (Figure 1). Subsequently, we conducted an analysis of concordance of allergic disease history among the monozygotic and dizygotic twins.

### 2.3. Survey

The medical records were reviewed and a physical exam was performed. Trained interviewers recorded the participants’ history of cardiometabolic disorders (hypertension, hyperlipidemia, type 2 diabetes, stroke, transient ischemic attack, and ischemic heart disease). The participants’ glycated hemoglobin (g/dL), low-density lipoprotein (mg/dL), high-density lipoprotein (mg/dL), triglyceride (mg/dL), total cholesterol (mg/dL), insulin (uIU/mL), and fasting blood glucose (mg/dL) levels were assessed. In addition, systolic blood pressure (mmHg) and diastolic blood pressure (mmHg) were also monitored. The participants’ monthly incomes were classified as non-respondent, low income (<$2000 per month), middle income ($2000–$3999 monthly), and high income (≥$4000 monthly). Educational level was classified as lower than high school, high school, or college level (dropped out or graduated from college). Marital status was categorized as unmarried, married, divorced, or other. Physical activity levels were classified as intense, moderate, walking time, or sitting time in either the workplace or at home. The participants’ body mass index (BMI) was calculated in kg/m^2^ using the health checkup data. Based on smoking history, the participants were classified into non-smokers (<100 cigarettes in their entire life), past smokers (quit for longer than 1 year), and current smokers. Based on alcohol consumption frequency, the participants were classified as non-drinkers and those who consumed alcohol ≤1 time per month, 2–4 times monthly, and ≥2 times weekly. Sleep time was measured on 5 weekdays and 2 weekends. Lastly, recent medication histories, including the usage of acetaminophen and aspirin, and non-steroidal anti-inflammatory drug usage ≥3 months were recorded.

### 2.4. Exposure

This study examined the two types of twins (monozygotic and dizygotic) as distinct groups. Individuals born in multiple births with more than two offspring were not included in the research.

### 2.5. Outcome

We gauged the similarity of occurrence of allergic conditions (asthma, allergic rhinitis, atopic dermatitis, and allergic conjunctivitis) between twin participants of a classical twin study, with the concordance rate being the main measure. A greater concordance between identical twins than fraternal twins would indicate a greater role for the genetic component over environmental factors; however, if there was a similar level of difference between the twin pairs in the two groups, it would likely be the result of environmental influence. The history of allergic disease in the twin pairs was split into positive-positive, positive-negative, or negative-negative. Twin pairs in which both siblings either had or did not have a disease or trait were classed as concordant (positive-positive and negative-negative, respectively).

### 2.6. Statistical Analyses

A chi-square test (for categorical variables) and Wilcoxon rank-sum test (for continuous variables) were used to analyze the baseline characteristics of participants. ORs and 95% confidence intervals (CI) were calculated to determine the concordance of allergic diseases. A binomial logistic regression model was employed to calculate the ORs of monozygotic twins ([positive-positive or negative-negative]/[positive-negative]) compared to dizygotic twins. In addition, a multinomial logistic regression model was utilized to calculate the ORs of monozygotic twins in comparison to dizygotic twins. For the evaluation of outcomes, a crude model, an adjusted Model 1 (including factors like age, sex, income, education level, marital status, physical activity level, obesity, smoking history, alcohol consumption frequency, sleep time, and medication histories) and an adjusted Model 2 (factors included in Model 1 plus histories of asthma, allergic rhinitis, atopic dermatitis, and allergic conjunctivitis) were used. Two-tailed analyses were conducted and statistical significance was set at *p* < 0.05. All the data were analyzed using the statistical package for the social sciences (version 24.0; IBM, Armonk, NY, USA).

## 3. Results

Table 1 provides a summary of the comparison of baseline characteristics between the monozygotic and dizygotic twins. The prevalence rates of allergic diseases, such as asthma, allergic rhinitis, atopic dermatitis, and allergic conjunctivitis, showed no significant difference between monozygotic and dizygotic twins (*p* = 0.346, *p* = 0.158, *p* = 0.314, and *p* = 0.227, respectively). There were significant differences in the sex ratio, distribution of age groups, and level of intense physical activity between monozygotic and dizygotic twins (*p* = 0.004, *p* = 0.015, and *p* = 0.013, respectively). However, other variables, such as education, income, physical activity levels (except the intense level), obesity, marital status, alcohol consumption frequency, smoking history, and sleep time, showed no significant difference between the two groups (all *p* > 0.05).

**Figure 2:**
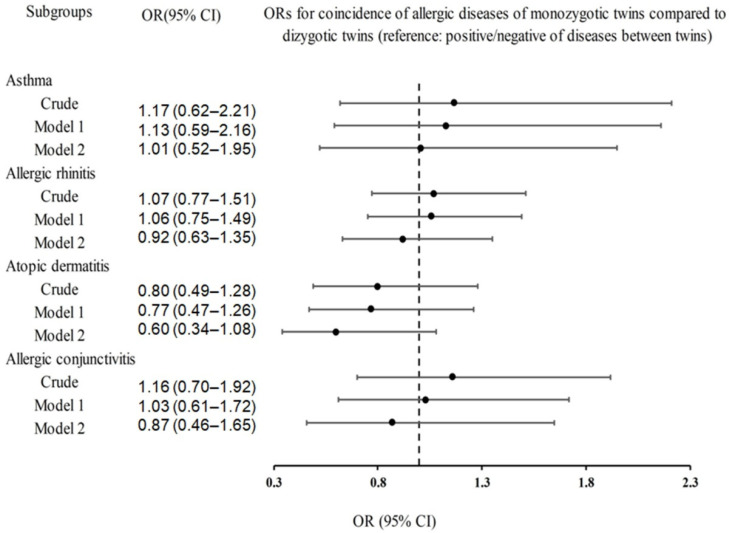
Forest plots for the ORs (95% CIs) of coincidence of asthma, allergic rhinitis, atopic dermatitis, and allergic conjunctivitis according to the crude model, Model 1 (adjusted for sex, age, income, obesity, education level, physical activity level, alcohol consumption frequency, smoking history, marital status, and sleep time), and Model 2 (adjusted for factors included in Model 1 plus adjustments for history of each disease [asthma, allergic rhinitis, atopic dermatitis, or allergic conjunctivitis]) analyses. Abbreviations: OR, odds ratio; CI, confidence interval.

We examined the agreement rates of the occurrence or non-occurrence of asthma, allergic rhinitis, atopic dermatitis, and allergic conjunctivitis in monozygotic twins in comparison to those in dizygotic twins (Table 2 and Figure 2). The concordance rate (92%) of atopic dermatitis in monozygotic twins was slightly higher than that in dizygotic twins (90.2%); however, the association showed only a borderline significance (*p* = 0.090). The concordance rates of other allergic diseases within monozygotic twins were lower compared to dizygotic twins (asthma 94.3% vs. 95.1%; allergic rhinitis 77.5% vs. 78.7%; allergic conjunctivitis 90.6% vs. 91.8%). The crude and adjusted ORs for the agreement rates of presence or absence of asthma, allergic rhinitis, atopic dermatitis, and allergic conjunctivitis in monozygotic twins were not significantly higher than those in dizygotic twins (*p* = 0.979, *p* = 0.670, and *p* = 0.674, respectively).

**Figure 3:**
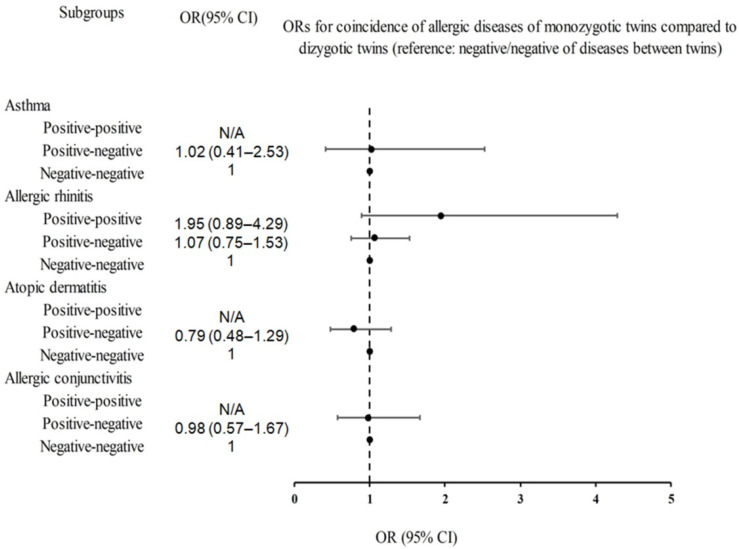
Forest plots for ORs (95% CIs) of whether the incidence of asthma, allergic rhinitis, atopic dermatitis, and allergic conjunctivitis were more common in monozygotic or dizygotic twins. Abbreviations: OR, odds ratio; CI, confidence interval.

We also investigated whether the prevalence of allergic diseases was higher within monozygotic or dizygotic twins (Table 3 and Figure 3). Monozygotic twins had a higher proportion of cases in which both siblings had allergic diseases compared to dizygotic twins (asthma, 1.1% vs. 0.0%; allergic rhinitis, 6.7% vs. 3.3%; atopic dermatitis, 2.9% vs. 0.0%; allergic conjunctivitis, 1.5% vs. 0.0%), but this was not statistically significant.

After adjusting for covariates, the likelihood of developing asthma, allergic rhinitis, atopic dermatitis, and allergic conjunctivitis in monozygotic twins was not significantly higher than that in dizygotic twins (all *p* > 0.05). While the crude model and Model 1 ORs for allergic rhinitis indicated a significantly higher incidence in monozygotic twins (crude OR 2.16, 95% CI = 1.02–4.57, *p* = 0.044; Model 1 OR 2.14, 95% CI = 1.00–4.57, *p* = 0.049, respectively), fully adjusted analyses did not show a significant difference (Model 2 OR 1.95; 95% CI = 0.89–4.29; *p* = 0.097).

## 4. Discussion

In twin studies conducted in Korea, a comprehensive analysis of asthma, allergic rhinitis, atopic dermatitis, and allergic conjunctivitis with full adjustments for lifestyle and environmental factors has not yet been carried out. This cross-sectional study, utilizing data from a validated adult Korean twin cohort, may suggest a lack of agreement of prevalence of asthma, allergic rhinitis, atopic dermatitis, or allergic conjunctivitis in monozygotic twins compared to dizygotic twins. Despite adjusting for multiple confounding factors and mutual effects of allergic diseases, we did not find any elevated probability of these allergic diseases in either twin group. Given the scarcity of validated twin cohort data, our present twin study may contribute to expanding our comprehension of the interplay between environmental factors and genetic contributions to the development of allergic diseases, such as asthma, allergic rhinitis, atopic dermatitis, or allergic conjunctivitis among the Korean population.

By comparing clinical parameters of a disease state in monozygotic twins to the corresponding measurements in dizygotic twins, it becomes feasible to generate evidence for the genetic basis of those parameters under reasonable assumptions [26]. Total concordance rates, which consider twin pairs as concordant if they both do or do not have the disease, have been proposed as a means of assessing the genetic influence on determining a specific trait or state of illness [26]. The absence of concordance in the phenotypic expression of asthma, atopic dermatitis, or allergic conjunctivitis among monozygotic twins might suggest the potential impact of environmental factors that influence disparate lifestyle behaviors in monozygotic twins who share the same genetic background, age, and sex. Although the concordance rate of atopic dermatitis in monozygotic twins (92%) was slightly greater than that in dizygotic twins (90.2%), this trend only exhibited borderline significance (*p* = 0.090), indicating a weak association that could be influenced by shared genetics.

Monozygotic twins had a higher proportion of cases in which both siblings had allergic diseases compared to dizygotic twins (asthma, 1.1% vs. 0.0%; allergic rhinitis, 6.7% vs. 3.3%; atopic dermatitis, 2.9% vs. 0.0%; allergic conjunctivitis, 1.5% vs. 0.0%). Regarding allergic rhinitis, its concordance was higher in monozygotic twins than in dizygotic twins in the crude model and in Model 1 after controlling for sociodemographic and clinical factors such as age, sex, obesity, smoking history, alcohol consumption frequency, income, education level, marital status, physical activity level, and sleep time. These results indicate that socioeconomic, demographic, and lifestyle factors might not significantly mediate the concordance of allergic rhinitis in monozygotic twins. Nonetheless, these associations became insignificant after accounting for the history of other allergic diseases such as asthma, atopic dermatitis, and allergic conjunctivitis, which might imply that the presence of comorbid allergic diseases could be a mediator of the concordant manifestation of allergic rhinitis in monozygotic twins. There is a frequent co-occurrence of allergic diseases in the same individual, with one disease being accompanied by another [27]. Recent studies have spotlighted on the imbalance of gut homeostasis and subsequent disruption of tolerance, which may have noteworthy clinical implications for the emergence of allergic diseases [28], since these allergic diseases might be associated with dysbiosis of the commensal microbiome, especially in the gut [28]. A clinical trial involving a twin study of allergic diseases has indicated that healthy twins and those with allergies, throughout their adulthood, presented significant variations in their gut microbiome composition and metabolomes within twin pairs [19]. These findings suggest that early-life circumstances, such as maternal and childhood nutrition, may have a protective role against the emergence of allergic diseases in adulthood [19]. Although monozygotic twins showed a tendency for a greater proportion of cases in which both siblings had allergic diseases than dizygotic twins, comorbidities seem to play a significant role in the development of allergic diseases in adult twins, and early-life interventions and health management may be targeted towards preventing their development.

There has been limited research comparing twin studies on allergic diseases in Korea with those in other countries. Some studies have compared concordance rates for asthma between monozygotic and dizygotic twins [16,18], and dissimilar clinical manifestations of asthma have been reported in identical twins as well [29]. However, there have been fewer twin studies of other allergic diseases such as allergic rhinitis, atopic dermatitis, or allergic conjunctivitis. A United Kingdom twin study based on 340 monozygotic and 533 dizygotic pairs, aged 18 to 72 years, has reported that genetically identical twins are often discordant in their expression of atopy, suggesting the significant influence environmental factors may have in disease expression [30]; these results are similar to those from our study. In a Chinese twin study [31], a large number of monozygotic twins were discordant in sensitization to common allergens of allergic diseases, suggesting that both genetic and environmental factors influence sensitization. 

Many studies have considered the possibility of a genetic predisposition being a contributing factor to the development of clinical allergic conditions [26,30,32]. However, the disparity in the manifestation of disease phenotype between monozygotic twin pairs could be partially attributed to epigenetic variations [33,34]. These variations might result in the mitigation of inherited traits for disease occurrence, primarily by regulating gene expression through the modification of chromatin accessibility to transcription factors [33]. Although twins may be epigenetically identical during the early stages of life, older monozygotic twins exhibit significant differences in the genomic distribution of 5-methylcytosine DNA and histone acetylation [35]. These differences could result in distinct genetic expression patterns and disease phenotypes as they age [19,30]. The discordant variation in disease expression between monozygotic twin pairs may be attributed to a combination of gene-environment interactions and epigenetic disparities. Indeed, a twin genetic study conducted on a younger population, aged 6 to 31 years, provided evidence for a greater genetic influence on the expression of allergic diseases than the influence of environmental factors [26]. However, a twin study conducted on a population aged 18 to 72 years showed that genetically identical twins often display discordance in their expression of atopy, implying a significant influence of environmental factors [30]. Similarly, as our study involved adults, it is possible that environmental factors influenced the heritability of these diseases. Therefore, the lack of concordance of allergic diseases in monozygotic twins compared to dizygotic twins may be due to different environmental influences or acquired factors. Hence, it can be inferred that the development and manifestation of allergic diseases in adult populations are influenced by both genetic and environmental factors.

The study has several strengths, including the use of prospective twin cohort data regularly validated by national statisticians, which enhanced the reliability of the findings. Additionally, the study comprehensively considered potential confounders among lifestyle and socioeconomic factors, which strengthened the comparisons between twin pairs. The adjustments made for lifestyle factors were crucial because they have been documented as possible risk factors for the studied allergic diseases. Finally, to our knowledge, this is the first large-scale twin study on allergic rhinitis, asthma, allergic conjunctivitis, and atopic dermatitis in the Korean population, adding to the literature on the topic.

This study has some limitations that need to be addressed. First, the effects of unmeasured confounders could not be perfectly excluded, even though the study adjusted for a considerable number of variables. Second, there may have been recall bias because of the retrospective interview of participants, despite the use of validated questionnaires to examine medical histories. Third, the cross-sectional study design does not confirm the causal relationship between twin births and allergic disorders. Fourth, the presence of a lower number of twin pairs with allergic diseases might limit the generalizability of the results, although the study included one of the largest twin participant samples. Fifth, the lack of data on serum immunoglobulin E concentrations, results of the prick tests or genetic data on related allergic diseases may be additional limitations. Sixth, we could not assess the non-twin siblings’ data as control patients, nor could we assess the concordance in the severity of the allergic diseases between monozygotic and dizygotic twins.

## 5. Conclusions

To summarize, the study did not find meaningful differences in the concordance rates of allergic diseases between monozygotic and dizygotic twins, indicating a limited role for genetics in the incidence of allergic diseases in the Korean adult population. The study suggests that environmental factors may have a more substantial contribution to the incidence of allergic diseases. However, the study has some limitations, including the possibility of unmeasured confounders and recall bias. Overall, further research is necessary to confirm these findings and to investigate the potential environmental factors contributing to the prevalence of allergic diseases in the Korean population.

## Figures and Tables

**Table 1:** General Characteristics of Participants.

Characteristics		Total Participants		
		Monozygotic Twin	Dizygotic Twin	*p*-Value
Age (years old, n, %)				0.004 *
	20–24	6 (0.6)	0 (0)	
	25–29	68 (6.5)	4 (1.6)	
	30–34	362 (34.4)	87 (35.7)	
	35–39	244 (23.2)	65 (26.6)	
	40–44	139 (13.2)	36 (14.8)	
	45–49	131 (12.4)	20 (8.2)	
	50–54	82 (7.8)	22 (9)	
	55–59	14 (1.3)	10 (4.1)	
	60–64	4 (0.4)	0 (0)	
	65+	2 (0.2)	0 (0)	
Sex (n, %)				0.015 *
	Males	386 (36.7)	110 (45.1)	
	Females	666 (63.3)	134 (54.9)	
Income (n, %)				0.984
	<2 million (won)	349 (33.2)	81 (33.2)	
	2 to <3 million (won)	284 (27.0)	68 (27.9)	
	3 to <4 million (won)	214 (20.3)	50 (20.5)	
	≥4 million (won)	205 (19.5)	45 (18.4)	
Education (n, %)				0.752
	Under high school	120 (11.4)	25 (10.2)	
	Graduated from High school	371 (35.3)	92 (37.7)	
	Commercial college-Dropped out of college	123 (11.7)	32 (13.1)	
	Graduated from High school	436 (41.5)	95 (38.9)	
Marriage (n, %)				0.325
	Unmarried	247 (23.5)	50 (20.5)	
	Married	738 (70.2)	173 (70.9)	
	Divorced or others	67 (6.4)	21 (8.6)	
Physical Activity				
	Hard (hour/1 week, mean, SD)	3.1 (6.8)	4.7 (9.7)	0.013 *
	Moderate (hour/1 week, mean, SD)	5.8 (10.5)	6.2 (10.2)	0.606
	Walk (hour/1 week, mean, SD)	6.1 (9.6)	6.8 (10.9)	0.299
	Sit (hour/1 week, mean, SD)	40.1 (22)	37.9 (20.7)	0.153
Obesity (n, %)				0.235
	Underweight (BMI < 18.5)	27 (2.6)	5 (2)	
	Normal (BMI ≥ 18.5 to <23)	510 (48.5)	113 (46.3)	
	Overweight (BMI ≥ 23 to <25)	221 (21)	68 (27.9)	
	Obese I (BMI ≥ 25 to <30)	262 (24.9)	52 (21.3)	
	Obese II (BMI ≥ 30)	32 (3)	6 (2.5)	
Smoking status (n, %)				0.151
	Nonsmoker	691 (65.6)	145 (59.4)	
	Past smoker	109 (10.4)	33 (13.5)	
	Current smoker	252 (24.0)	66 (27.0)	
Frequency of drinking alcohol (n, %)				0.326
	Nondrinker	304 (28.9)	64 (26.2)	
	≤1 time per month	238 (22.6)	46 (18.9)	
	2–4 times per month	301 (28.6)	80 (32.8)	
	≥2 times per week	209 (19.9)	54 (22.1)	
Sleeping hours (n, %)				0.388
	≤5 h	54 (5.1)	16 (6.6)	
	6–7 h	620 (58.9)	146 (59.8)	
	8–9 h	350 (33.3)	72 (29.5)	
	≥10 h	28 (2.7)	10 (4.1)	
Asthma (n, %)		42 (4.0)	6 (2.5)	0.346
Allergic rhinitis (n, %)		189 (18.0)	34 (13.9)	0.158
Atopic dermatitis (n, %)		72 (6.8)	12 (4.9)	0.314
Allergic conjunctivitis (n, %)		66 (6.3)	10 (4.1)	0.227
Acetaminophen prescription ≥ 3 months (n, %)		44 (4.2)	6 (2.5)	0.268
Aspirin prescription ≥ 3 months (n, %)		33 (3.1)	3 (1.2)	0.129
NSAID prescription ≥ 3 months (n, %)		18 (1.7)	8 (3.3)	0.128

* Significance at *p* < 0.05, chi-square test (categorical variables) or Wilcoxon rank-sum test (continuous variables) was performed. Abbreviations: SD, standard deviation, BMI, body mass index, NSAID, non-steroidal anti-inflammatory drug.

**Table 2:** Analysis of odds ratios with 95% confidence interval of coincidence of allergic diseases in monozygotic twins compared to that in dizygotic twins (reference: positive/negative results for diseases between twin siblings).

Coincidence of Diseases		Monozygotic Twin	Dizygotic Twin	Odds Ratios					
		n (%)	n (%)	Crude	*p*-Value	Model 1 *	*p*-Value	Model 2 †	*p*-Value
Asthma									
	concordant	992/1052 (94.3)	232/244 (95.1)	1.17 (0.62–2.21)	0.630	1.13 (0.59–2.16)	0.711	1.01 (0.52–1.95)	0.979
	discordant	60/1052 (5.7)	12/244 (4.9)	1		1		1	
Allergic rhinitis									
	concordant	815/1052 (77.5)	192/244 (78.7)	1.07 (0.77–1.51)	0.681	1.06 (0.75–1.49)	0.756	0.92 (0.63–1.35)	0.670
	discordant	237/1052 (22.5)	52/244 (21.3)	1		1		1	
Atopic dermatitis									
	concordant	968/1052 (92)	220/244 (90.2)	0.80 (0.49–1.28)	0.347	0.77 (0.47–1.26)	0.300	0.60 (0.34–1.08)	0.090
	discordant	84/1052 (8)	24/244 (9.8)	1		1		1	
Allergic conjunctivitis									
	concordant	953/1052 (90.6)	224/244 (91.8)	1.16 (0.70–1.92)	0.554	1.03 (0.61–1.72)	0.918	0.87 (0.46–1.65)	0.674
	discordant	99/1052 (9.4)	20/244 (8.2)	1		1		1	

* Adjusted for age, sex, income, education level, marital status, physical activity level, obesity, smoking history, alcohol consumption frequency, sleep time, and medication histories. † Factors included in Model 1 plus histories of each disease (asthma, rhinitis, atopy, and conjunctivitis). “Concordant” indicates concordant positive-positive or negative-negative results between monozygotic or dizygotic twin siblings, whereas “discordant” indicates discordant positive and negative results between monozygotic or dizygotic twin siblings.

**Table 3:** Analysis of odds ratios with 95% confidence intervals of concordance of allergic diseases in monozygotic twins compared to that in dizygotic twins (reference: negative/negative results for diseases between twin siblings).

Concordance of Diseases		Monozygotic Twin	Dizygotic Twin	Odds Ratios (95% CI)					
		n (%)	n (%)	Crude	*p*-Value	Model 1 *	*p*-Value	Model 2 †	*p*-Value
Asthma									
	Positive-positive	12/1052 (1.1)	0/244 (0)	N/A	N/A	N/A	N/A	N/A	N/A
	Positive-negative	60/1052 (5.7)	12/244 (4.9)	1.18 (0.63–2.24)	0.603	1.14 (0.60–2.18)	0.690	1.02 (0.41–2.53)	0.97
	Negative-negative	980/1052 (93.2)	232/244 (95.1)	1		1		1	
Allergic rhinitis									
	Positive-positive	70/1052 (6.7)	8/244 (3.3)	2.16 (1.02–4.57)	0.044 *	2.14 (1.00–4.57)	0.049*	1.95 (0.89–4.29)	0.097
	Positive-negative	237/1052 (22.5)	52/244 (21.3)	1.13 (0.80–1.58)	0.496	1.11 (0.79–1.57)	0.557	1.07 (0.75–1.53)	0.697
	Negative-negative	745/1052 (70.8)	184/244 (75.4)	1		1		1	
Atopic dermatitis									
	Positive-positive	30/1052 (2.9)	0/244 (0)	N/A	N/A	N/A	N/A	N/A	N/A
	Positive-negative	84/1052 (8)	24/244 (9.8)	0.82 (0.51–1.32)	0.417	0.80 (0.49–1.31)	0.375	0.79 (0.48–1.29)	0.514
	Negative-negative	938/1052 (89.2)	220/244 (90.2)	1		1		1	
Allergic conjunctivitis									
	Positive-positive	16/1052 (1.5)	0/244 (0)	N/A	N/A	N/A	N/A	N/A	N/A
	Positive-negative	99/1052 (9.4)	20/244 (8.2)	1.18 (0.72–1.96)	0.511	1.05 (0.63–1.76)	0.854	0.98 (0.57–1.67)	0.927
	Negative-negative	937/1052 (89.1)	224/244 (91.8)	1		1		1	

* Adjusted for age, sex, income, education level, marital status, physical activity level, obesity, smoking history, alcohol consumption frequency, sleep time, and medication histories. † Adjusted for factors included in Model 1 plus for histories of each disease (asthma, rhinitis, atopy, and conjunctivitis).

## Data Availability

All data are available from the database of the National Health Insurance Sharing Service (NHISS) (https://nhiss.nhis.or.kr; accessed on 1 July 2022). NHISS allows access to all of these data for any researcher who promises to follow the research ethics at some processing charge. If you want to access the data of this article, you can download it from the website after promising to follow the research ethics.
